# On flattening filter‐free portal dosimetry

**DOI:** 10.1120/jacmp.v17i4.6147

**Published:** 2016-07-08

**Authors:** Eduardo Pardo, Juan Castro Novais, María Yolanda Molina López, Sheila Ruiz Maqueda

**Affiliations:** ^1^ S^o^ Radiofísica y Protección Radiológica, Hospital Universitario Quirón Madrid, Madrid, España (Medical Physics and Radiation Protection Department Quiron University Hospital Madrid Spain

**Keywords:** FFF, IMRT, VMAT, EPID, QA.

## Abstract

Varian introduced (in 2010) the option of removing the flattening filter (FF) in their C‐Arm linacs for intensity‐modulated treatments. This mode, called flattening filter‐free (FFF), offers the advantage of a greater dose rate. Varian's “Portal Dosimetry” is an electronic portal imager device (EPID)‐based tool for IMRT verification. This tool lacks the capability of verifying flattening filter‐free (FFF) modes due to saturation and lack of an image prediction algorithm. (Note: the latest versions of this software and EPID correct these issues.) The objective of the present study is to research the feasibility of said verifications (with the older versions of the software and EPID). By placing the EPID at a greater distance, the images can be acquired without saturation, yielding a linearity similar to the flattened mode. For the image prediction, a method was optimized based on the clinically used algorithm (analytical anisotropic algorithm (AAA)) over a homogeneous phantom. The depth inside the phantom and its electronic density were tailored. An application was developed to allow the conversion of a dose plane (in DICOM format) to Varian's custom format for Portal Dosimetry. The proposed method was used for the verification of test and clinical fields for the three qualities used in our institution for IMRT: 6X, 6FFF and 10FFF. The method developed yielded a positive verification (more than 95% of the points pass a 2%/2 mm gamma) for both the clinical and test fields. This method was also capable of “predicting” static and wedged fields. A workflow for the verification of FFF fields was developed. This method relies on the clinical algorithm used for dose calculation and is able to verify the FFF modes, as well as being useful for machine quality assurance. The procedure described does not require new hardware. This method could be used as a verification of Varian's Portal Dose Image Prediction.

PACS number(s): 87.53.Kn, 87.55.T‐, 87.56.bd, 87.59.‐e

## I. INTRODUCTION

### A. Intensity‐modulation verification

It is a well‐known fact that, in recent years, the use of intensity‐modulated radiotherapy (IMRT) and its rotational counterpart, volumetric modulated arc therapy (VMAT), has become widespread. Intensity‐modulated techniques, either static or rotational, optimize dose distributions by creating different radiation intensity levels at different points inside a field. This is achieved through multileaf collimator (MLC) movement during irradiation (sliding windows) (with or without gantry rotation) or administering several small fields (step and shoot) (both small in size and monitor units).

Every RT treatment plan should undergo some kind of verification prior to treatment. For 3D treatments, usually a standard machine QA plus an alternative calculation is considered sufficient. Standard monthly machine QA might not guarantee that an IMRT plan is correctly delivered and that is why per‐patient measurements are usually done. Not only absolute point dose but also relative dose distribution should be verified.^1^, ^2^, ^3^, ^4^

The verification of IMRT fields needs the measurement of dose distributions verifying the treatment planning system (TPS) calculation and the capacity of the system to administer it. Two main approaches are found: measurement of the field in a plane perpendicular to its axis (fluence measurement), and measurement of a dose plane on a phantom (where all the ports have their real gantry angles). These dose distributions are later compared with the prediction from the TPS (on the same conditions).

The most commonly used dose distribution measurement devices currently available are film^5^, ^6^ (radiochromic), arrays^7^, ^8^ (both ion chamber and diodes), and EPIDs. Film has a high resolution, but its use is very time‐consuming; it needs time before scanning, the scanning itself, and a calibration process. Ionization chamber arrays usually lack the needed resolution, both in measurement volume and chamber distance. Diode arrays have almost point‐like size but interspace is usually in the cm scale (for most commercial models available). These resolutions might be too low for the verification of plans designed for MLCs with 2.5 mm wide leafs.

Portal vision can be used for IMRT verification combining the strong points of both methods described earlier: speed and resolution. The images from the EPID are available (almost) immediately (don't need additional postprocessing) and offer an effective resolution below 0.5 mm. Portal vision also adds an extremely easy and fast setup. As a weak point, EPID does not have the capability of measuring a dose plane in a phantom and is limited to measuring the dose (fluence) on a plane perpendicular to the field's axis. Most modern portal systems are diode‐based (amorphous silicon). That means (even with the compensator Cu layer) that low energy photons are over‐detected.^9^, ^10^, ^11^, ^12^, ^13^, ^14^

### B. Portal dosimetry

Varian's “Portal Dosimetry” (Varian Medical Systems VMS, Palo Alto, CA) is an application integrated on the ARIA suite that allows IMRT (and VMAT) field verification. It is composed of three steps: fluence prediction (inside the TPS, Eclipse, using the Portal Dose Image Prediction (PDIP) algorithm), fluence acquisition (using dosimetry mode on the linac), and fluence comparison (on the Portal Dosimetry module itself). It allows only the verification of dose modulated fields; it does not allow the verification of static or unflattened fields. (Note: the latest version of the software, version 13, used with the latest EPID, as1200 with DMI detector, can overcome these limitations.) Portal Dosimetry adds an extremely fast and easy workflow for the verification of intensity‐modulated fields.

### C. FFF modes

Conventional linacs produce X‐rays by bremsstrahlung, stopping high‐energy electrons with a high‐Z target. The photon distribution created is peaked; it has higher intensity on the center than on the borders. Most linacs use a flattening filter that creates a flattened distribution (and is responsible for the horns at certain depths). The flattening filter reduces the beam intensity between two and fourfold. Tomotherapy^15^, ^16^ and GammaKnife^(17)^ (Accuray Inc., Sunnyvale, CA) machines have been operating without a flattening filter for a long time, but only recently classical design machines have been researched on a FFF mode.^(18)^

In March 2010, the True Beam LINAC (VMS) introduced clinically the flattening filter‐free modes (FFF), later introduced on the Trilogy (also VMS) platform. The removal of the flattening filter has been talked about for a few years.^(18)^ This mode provides lower mean energy (increased skin dose) and greater dose rate (between two and fourfold, up to 2400 Mu/min on 10 FFF). These modes are suitable for high‐dose treatments (such as stereotactic body radiotherapy (SBRT) and stereotactic radiosurgery (SRS)). On gated treatments, the high dose rate can also be an advantage. Varian's flagship is called True Beam to honor the FFF modes.^19^, ^20^, ^21^, ^22^

The lack of field flatness is not a problem for intensity‐modulated fields because intensity is not flat anyway, and the beam profile can be accounted for during inverse optimization.

There is no Portal Dosimetry available for the FFF modes (as noted before prior to version 13 and without the new AS1200 EPID). The EPID application does not allow calibration for those modes and the high‐dose rates produce EPID saturation*.

Some research has been done to allow the verification of such fields like the work of Min et al.^(23^) that converts the plan to a lower dose rate to allow the verification.

An algorithm, called GLAaS^(24)^, has been used as an alternative to Varian's PDIP to verify dose distributions with EPID. GLAaS models the direct and transmitted (through MLC) radiation separately, to account for the different spectra and the overresponse of the EPID to low‐energy photons transforming the acquired image into a dose plane in water. Nicolini et al.^24^, ^25^ verified the GLAaS algorithm for FFF beams. (A comparison between the GLAaS algorithm and the proposed method can be seen in the Discussion section.)

In this work, we analyze the feasibility of using Portal Dosimetry for unflattened modes and we present a method for fluence prediction that actually uses the clinical dose algorithm (AAA). The whole method keeps the Portal Dosimetry as the comparison tool and only adds a few minutes (per field) to the verification. As a bonus, the method presented can be used for machine QA.

## II. MATERIALS AND METHODS

For all the measurements a Varian TB STX 1.6 (VMS) was used. The EPID is an AS1000 model. This amorphous silicon‐based EPID has a resolution of 1024×768 and a physical size of 30×22 cm (yielding a 0.384 mm pixel size). The images are automatically projected to source detector distance SDD=100 by the system, so this resolution is effectively increased if the EPID is set at a distance greater than 100.

### A. Dosimetry mode calibration

The EPID can work on a special mode called dosimetry mode. This mode acquires frames during irradiation. There is no synchronization of the linac and EPID^†^ so the irradiation happens undisturbed. This lack of synchronization causes horizontal lines when the number of monitor units is small due to the EPID being read while it is receiving a radiation pulse. If the amount of radiation between two frames is high enough (around 4–5 MU), there will be saturation and information will be lost.

The mode calibration is composed of four parts: dark field, flood field, pixel defect map, and absolute dose calibration. Dark field is an acquisition without radiation (obviously its SSD is irrelevant). Flood field is an acquisition of a field that covers the whole detector; on the True Beam platform this acquisition is done at SDD 100. Since the linac's profile is not completely flat (it has horns), the beam profile is introduced to correct for the different dose delivered to different portions of the EPID.

Using the information of the previous images (dark and flood fields), the system computes pixel defect map and corrects it by averaging the neighboring pixels. The last step is absolute dose calibration. This is the acquisition of a 10×10 field with a known number of monitor units (usually 100 MU), which are assigned a value in what is called Calibration Units (CU) (usually 100 MU will be assigned 100 CU).

The system does not allow these steps to be performed for the unflattened modes, but allows the acquisition of images with the corrections of the flattened field of the same energy (as noted previously, TrueBeam 2.0 overcomes this limitation).

Since absolute dose cannot be calibrated, the resulting CUs will have an arbitrary value for FFF mode; there will not be a user‐calibrated relation, but the value will be constant over time (for TrueBeam 2.0, this step can actually be carried leaving the rest of the procedure unchanged).

### B. Feasibility of FFF verification

The reason for the lack of FFF Portal Dosimetry was that the EPID would saturate. To test this we delivered several fields with several monitor units and dose rates to check the linearity of the system for both of the unflattened modes (6FFF and 10FFF) and the conventional 6X mode. EPID's signal was measured as the average of pixel value in the center of the field; this was done using a tool from portal dosimetry: “output factor tool”.

Also different SDDs were explored. A greater SDD means a worse signal‐to‐noise ratio (SNR) and greater effective resolution. Both factors are quite irrelevant, because resolution is already very good (well on the submillimeter scale) and FFF plans are likely to have a great number of monitor units (to take advantage of the high dose rate) that guarantee a good SNR. An increased SSD also places a limit on the maximum field size; this should not be a great restriction for FFF fields that are usually small (SBRT and SRS), so field size will most likely not pose a limitation.

After checking the feasibility of the image acquisition, the fluence prediction was developed.

### C. Fluence prediction

To generate the predicted image of the EPID, the dose plane calculated on a homogeneous phantom was used. This dose plane was calculated using Eclipse's clinical AAA(^26^) algorithm. The image is exported in DICOM‡ format (and converted to Varian's custom format “.dxf”. The “.dxf” format is a text format with a header of demographics and a description of the file's content. The only manipulation done to the data is rescaling from pixel value to CU. The .dxf file can be imported on the Portal Dosimetry workspace as a predicted image.

The homogeneous phantom used was tailored to match EPID's image. Only two parameters were empirically chosen: depth and CT number. The latter was chosen at a high value to mimic the EPID's material (and its overdetection of low‐energy photons). The chosen value is 3000 HU. As for the depth, it was chosen to match the beam profile of the 6X beam (the unflattened beam profile does not change as much with depth). The chosen depth is 3 cm. This yields an effective depth of 6.7 cm (roughly agreeing with the value of 7 cm used by Khan et al.^(9^) for its initial model).

### D. Data format conversion

One of Portal Dosimetry's main advantages is that it is fast and easy to use. To make this method also fast and easy to use, the framework of the Portal Dosimetry application had to be retained. For this purpose, an in‐house application was developed^§^.

**Figure 1:**
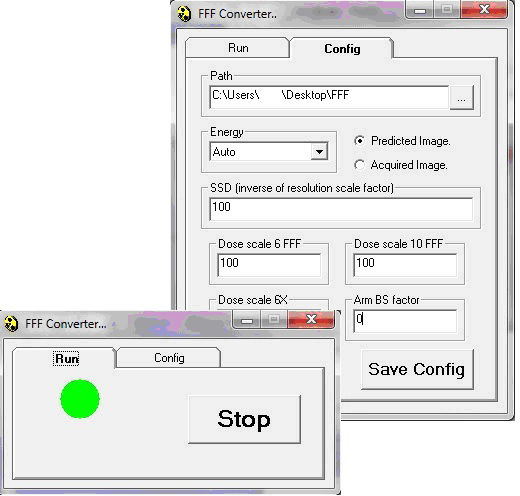
The application developed for the conversion of DICOM dose planes to .dxf format. Waiting for DICOM dose planes (left) and configuration tab (right).

The developed application (Fig. 1) monitors certain folders for DICOM files and automatically performs the file conversion as the DICOM files are exported from the TPS (once the file is converted, the software changes its extension to signal that the conversion has already been done). This application is composed of two parts. The first is a Visual Basic (Microsoft Inc., Redmont, WA) program that “listens” for DICOM files and, when it finds them, calls a second part. The second part is a Java (Oracle, Redwood Shores, CA) class that leverages ImageJ's (public domain software) capability to extract the pixel value from the DICOM file (the dose plane) and write it (pixel by pixel) into a complete .dxf file (including the header with mock data). There is no data manipulation beyond reescalation of pixel value to match the value of CUs in the acquired image. This rescaling factor allows for the absolute value of CU to be precisely adjusted to account for the linac's output or the correction for output factor of the precise field size (see the Results section).

The application (once set in place) acts without interaction with the user.

### E. Field verification workflow

After plan creation the “verification plan” must be created. Instead of choosing portal dose (that is not available for FFF plans), calculation over a phantom must be chosen. The plan is calculated (field by field) on the homogeneous phantom (with a grid resolution of 1 mm, the minimum available). The dose plane is exported in DICOM format to a specific folder. A placeholder plan must be created and assigned integrated (dosimetry) images (SDD of 160 should be checked also).

**Figure 2:**
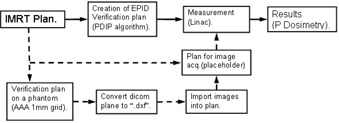
Workflow of Portal Dosimetry verification. Solid line, Varian (PDIP); dashed line, proposed method.

From the Portal Dosimetry workspace, the .dxf file (automatically generated by the previously described application) can be imported into the placeholder plan. The rest of the verification should go like it does for the regular Portal Dosimetry verifications: acquisition on the linac and comparison on the Portal Dosimetry workspace. The whole process only adds a few (3−5) minutes per field and can be carried out by a trained radiographer. The whole process, compared with the original Varian procedure, is shown in Fig. 2.

### F. Test cases

This method was tested (for the three qualities used in our hospital for IMRT (or VMAT) 6X, 6FFF and 10FFF) with: square fields, test fields (ziggurat and checkered), and clinical cases.

The square fields were tested in absolute dose mode to check the ability of the system to predict the different field sizes (output factors) in absolute dose (after calibration) and profile. The square fields were collimated with the MLC (with the jaws retracted 1 cm, except for the 1×1 and 2×2 cm, which were retracted 5 mm).

**Figure 3:**
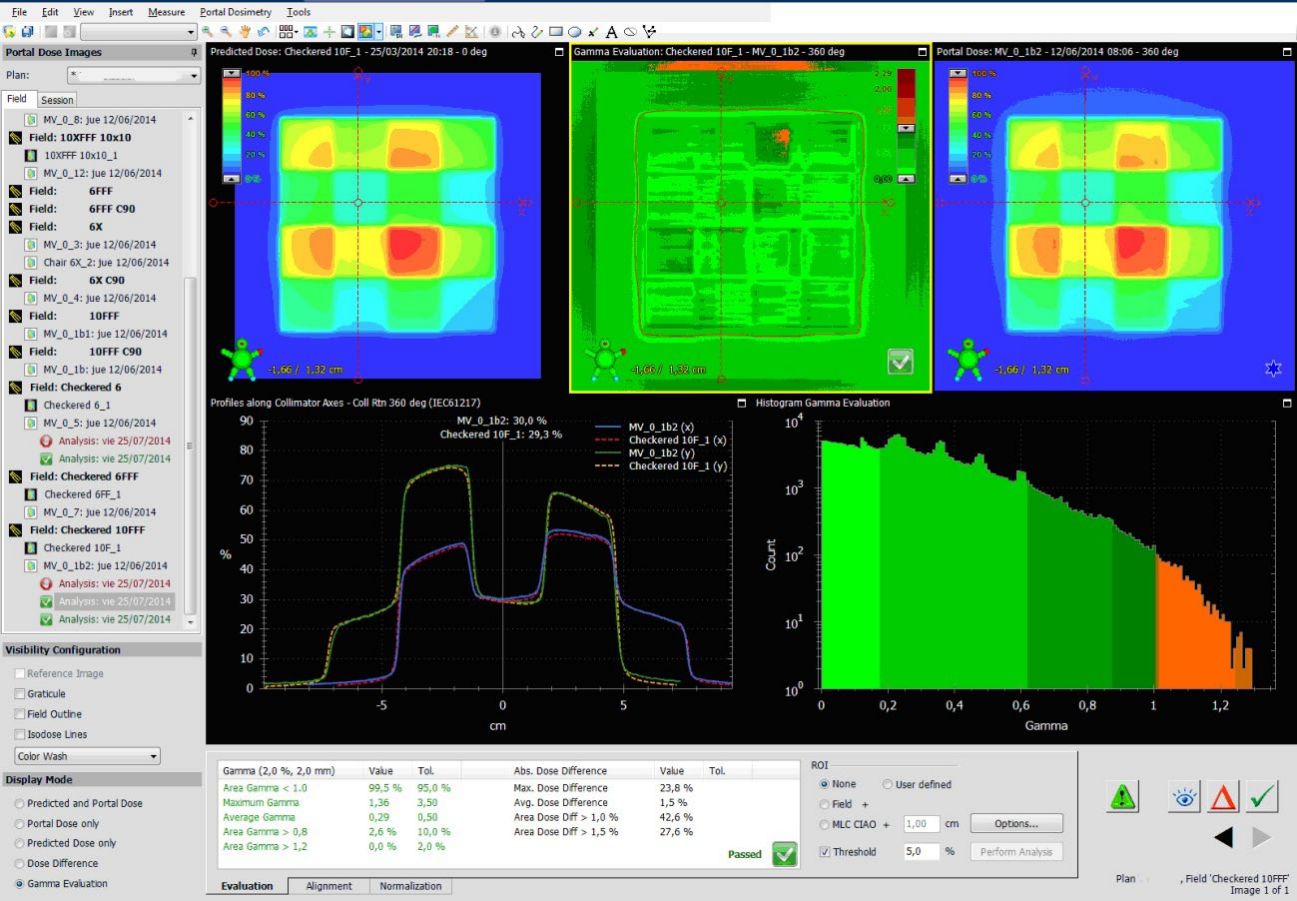
Test case: checkered field 10FFF.

**Figure 4:**
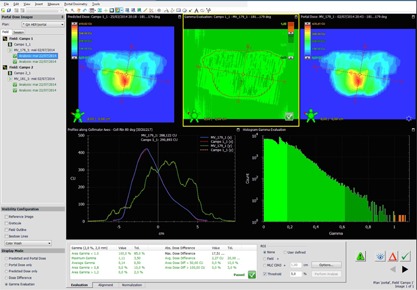
Example of a VMAT 10FFF field.

The test fields consist of two fields: ziggurat and checkered field (see Figs. 3 and 4).These constitute a quite extreme case of intensity‐modulation. These fields underwent the exact verification that is done in our institution for clinical fields before treatment (Table 1), that is, Portal Dosimetry evaluation in relative mode (although absolute is also checked)^**^, global gamma^27^, ^28^ 2%/2 mm with a threshold of 5% of maximum dose. A field is clinically acceptable if at least 95% of its points get a score gamma under 1 (pass the gamma test). Other parameters are checked to detect problems on the field, but are not used as a pass‐fail test (parameters such as gamma map, average gamma, maximum gamma, area gamma >0.8 and area gamma >1.2).

**Table 1:** Clinical fields verified

*Energy*	*Mode*	*Number of Fields*
6X	VMAT	4
6X	IMRT	9
6FFF	VMAT	6
10FFF	VMAT	65
10FFF	IMRT	9

The clinical cases are some randomly chosen cases representative of clinical treatments actually delivered at our hospital. For the 6X fields the verification with Varian's PDIP is also included. Most of the fields are 10FFF and VMAT because this is the beam quality most used for SBRT and SRS in our institution.

All of the clinical fields have also undergone absolute dose verification with an ionization chamber on a RW3 phantom (PTW, Freiburg, Germany) (results not included).

## III. RESULTS

### A. Feasibility

#### A.1 Signal vs. distance

**Figure 5:**
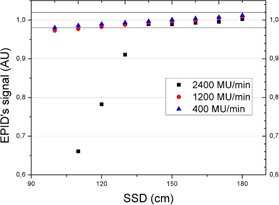
EPID signal vs. distance for three different dose rates. Saturation is seen on the higher dose rate, 2400 MU/min.

The signal from the EPID decreases with distance squared. Figure 5 shows the signal vs. distance (normalized).

Figure 5 shows that there is no EPID saturation (even for the highest available dose rate, 10FFF 2400 MU/min) beyond an SDD of 140 cm. For the clinical verifications an SDD of 160 cm was chosen to be far from the limit.

### B. Linearity

**Figure 6:**
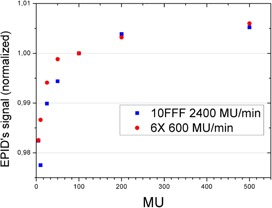
Comparison of linearity of 6X and 10FFF (both at their respective maximum dose rates: 600 MU/min and 2400 MU/min). Linearity (lack of) is below 1% above 20 MU.

**Figure 7:**
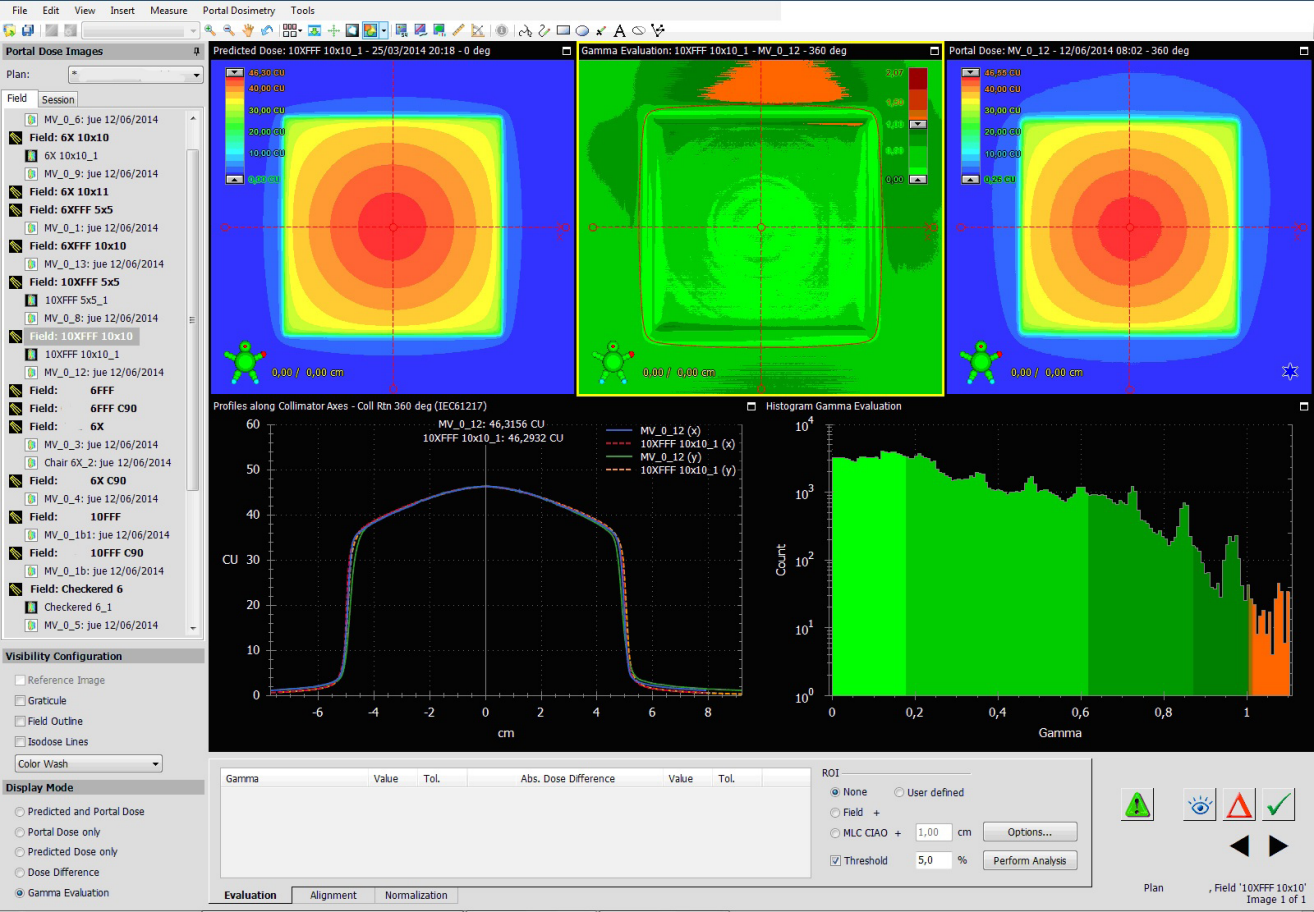
The Portal Dosimetry workspace during the analysis of a static square 10FFF field.

The linearity of EPID's response was also checked for the FFF modes and compared with 6X mode. Figure 6 shows a comparison between linearity for the highest dose rate mode (10FFF 2400 MU/min and the reference mode 6X). This figure shows that linearity is better than 1% above 20 MU. There is no difference between linearity on 6X and 10FFF or 6FFF (figure not shown). After proving that there is not significant saturation and that linearity is like the one found on 6X the verification of FFF modes was deemed feasible. The analysis of a square field 10FFF is shown in Fig. 7.

### C. Test cases

#### C.1 Output factors

**Figure 8:**
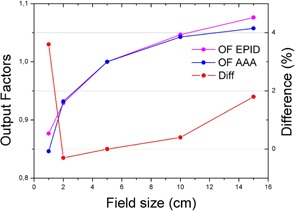
Comparison of the output factors (OF) as predicted by the TPS, Eclipse, and measured with the EPID for 10FFF. For fields between 2 and 10 cm, the output factors agree within 1%.

**Figure 9:**
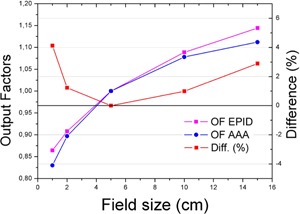
Comparison of the output factors (OF) as predicted by the TPS, Eclipse, and measured with the EPID for 6X. For fields between 2 and 10 cm, the output factors agree within 1.5%.

The phantom's depth was chosen to produce a beam profile that matched the acquired one; therefore, square fields were not a good test because they had been tailored that way. The output factors have been proved to be different between EPID and water.^(9)^ In our case, the output factors showed a good enough agreement (around 1%) for fields between a 2×2 cm and 10×10 (see Figs. 8, 9 and Table 2); this covers most clinical fields that are foreseeable for FFF. The normalization point was chosen at a field size of 5×5 cm (FFF fields are expected to be smaller because its main use will be SBRT and SRS).

**Table 2:** Output factors comparison between EPID and proposed method

	*Field Size*				
*Energy*	*1*	*2*	*5*	*10*	*15*
6X	4.12%	1.23%	0.00%	0.99%	2.90%
6FFF	3.50%	1.70%	0.00%	−0.91%	0.12%
10FFF	3,60%	−0.30%	0.00%	0.36%	1.76%

If a field should be verified and the difference in output factors is known to be bigger than desirable (e.g., for the verification of a 2×2 field with a gamma 1%/1 mm), the conversion factor from dose (on the DICOM image) to CU should be adjusted.

#### C.2 Nonclinical fields

The nonclinical fields are the ziggurat and the checkered fields (Fig. 3). Both fields test resolution and linearity. The six fields pass the verification (see Table 3). In the case of the 6X, the verification scores achieved by the proposed method are higher than the ones achieved by the PDIP. Clinical cases: 6X the scores were significantly better than the ones achieved with PDIP.

In Fig. 4, a verification of a 10FFF VMAT field can be seen.

For the results of the clinical verification for 6FFF (6 VMAT fields), see Table 4. For the results for 6X (9 IMRT fields and 4 VMAT fields) and 10FFF (65 VMAT fields and 9 IMRT fields), see Tables 5 and 6, respectively.

**Table 3:** Results of the verification of the test fields: ziggurat and checkered

*Field*	*Energy*	*Average Gamma*	*% Points Pass Gamma* 2%/2 mm ^a^
Ziggurat	6X	0.22	100
Checkered	6X	0.38	97.5
Ziggurat	6FFF	0.34	98.0
Checkered	6FFF	0.46	95.3
Ziggurat	10FFF	0.38	96.6
Checkered	10FFF	0.29	99.5

a ^a^ Gamma 2%/2 mm is the standard for dose distribution comparison on our hospital.

**Table 4:** Results of the clinical verification for 6FFF

	*MU*	*% Gamma Pass*	*Average Gamma*
mean	948	99.92	0.13
max	1082	100	0.18
min	834	99.5	0.1
median	954.5	100	0.12

**Table 5:** Results of the clinical verification for 6X

		*Proposed Method*		*PDIP*
	*MU*	*Gamma Pass*	*Av. Gamma*	*Gamma pass*		*Av. Gamma*
mean	314.31	97.58	0.26	93.29		0.39
max	810	100	0.5	100.00		0.66
mm	44	86.7	0.14	76.50		0.18
median	200	99.2	0.25	95.90		0.43

**Table 6:** Results of the clinical verification for 10FFF

	*MU*	*% Gamma Pass*	*Average Gamma*
mean	1118.88	99.81	0.17
max	3341	100.00	0.35
min	284	98.00	0.09
median	969	100.00	0.15

## IV. DISCUSSION

The presented method is capable of creating predicted images for Portal Dosimetry taking advantage of Aria's framework, although comparison through a third‐party application is also possible.

The method shows good promise for the comparison of predicted and measured images using absolute gamma; this will be the topic for future discussion. The presented method adds the advantage of using the clinical dose calculation algorithm being useful in the verification of the plan as a whole (without heterogeneity corrections). Moreover the setup time is almost zero (the algorithm is already configured).

PDIP uses a custom algorithm (pencil‐beam‐based) that needs modeling. Although the data needed for modeling are easy to acquire, it does add to the setup process. The fact that the calculation is based on different data than the clinical algorithm makes the method less useful for the verification of the plan, although it verifies the ability of the machine to deliver the planned fields.

**Figure 10:**
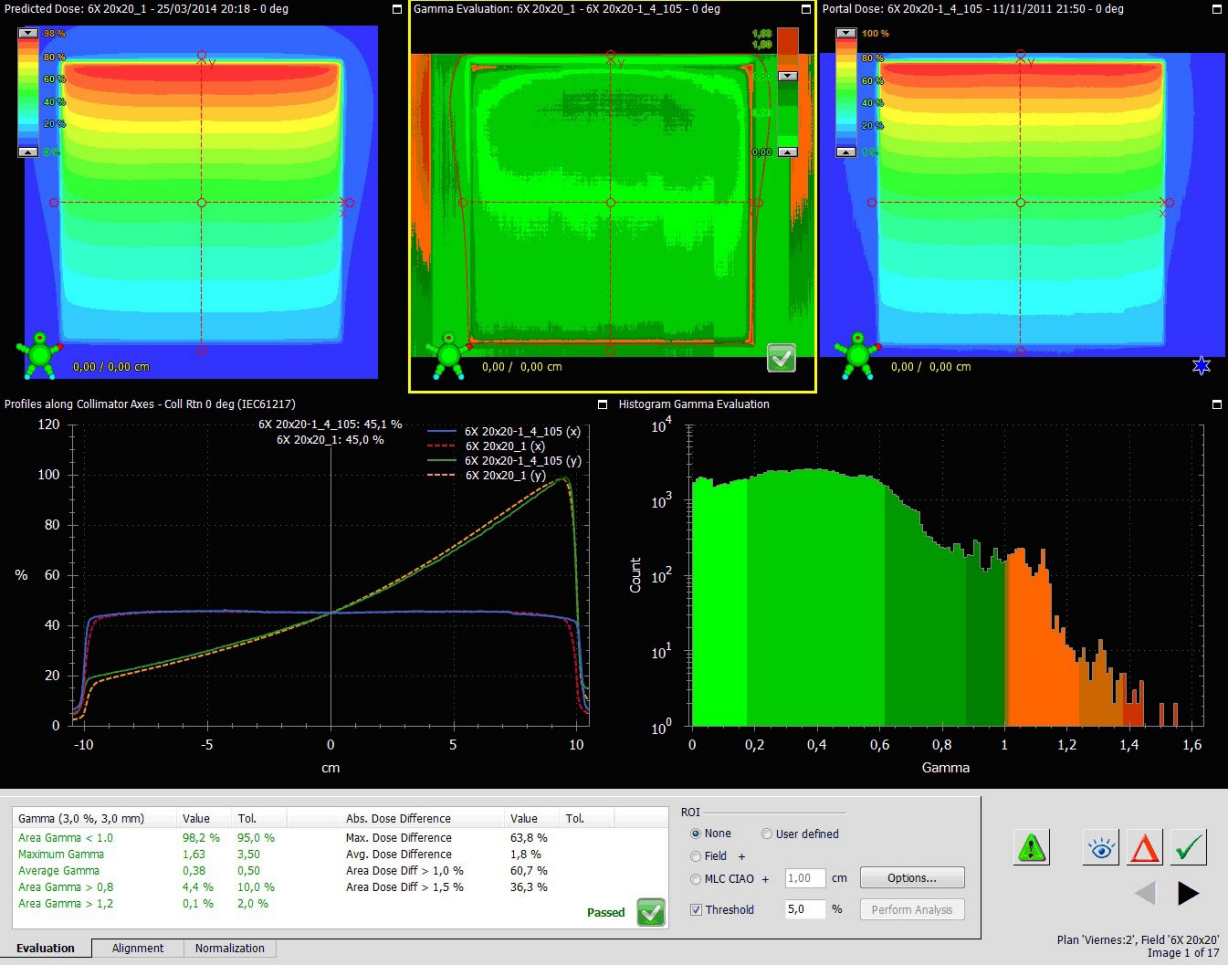
Proof of concept of the verification of a wedged field.

The method presented allows for the verification of any field that can be calculated with the AAA algorithm, making it able to do machine QA such as wedge verification (see Fig. 10).

The GLAaS method is similar to the one presented here, with the main difference being that the GLAaS algorithm makes the comparison on a water‐equivalent plane while the other (the one presented) makes the comparison on the EPID plane. None use kernel convolution to model EPID response; the calculation at a certain depth and the dose grid used for the calculation act as a convolution kernel that takes into account the detector response. The presented method uses a high #CT (3000 HU) instead of separating the direct and transmitted components. The method presented has the additional advantage of retaining the Portal Dosimetry framework.

EPID's support arm (called e‐arm) causes backscatter that makes beam profiles slightly slanted on the in‐plane direction.^(29)^ This backscatter is not the same for different field sizes. The proposed method could be used to slant the predicted image with a lineal function by a different amount for different field sizes. This will be the focus of future study.

All the calculations done are “open source”, this lets the user understand all the steps taken, troubleshoot problems, and add corrections, like the previously cited arm backscatter.

Table 7 shows a comparison of some key features of the method proposed and Varian's PDIP.

PDIP dosimetry system calculates a predicted image on the EPID, instead of a dose plane. The presented method assumes that the EPID image is comparable to a dose plane on a homogeneous phantom. The PDIP algorithm convolves the fluence with a set of 10 Gaussians of different sizes (although Van Esch and colleagues^(11)^ suggested only 3). According to the Van Esch study, the major contribution (95%) is due to the “narrow component”: less than one mm; 4% is due to the “few mm” component, and only 1% due to the long‐range scatter (“several cm”). The proposed method is calculated with a 1 mm dose grid that effectively takes into account the “narrow component” (under 1 mm) that makes up for the 95%. The other two components are taken into account by the scatter calculation done by the AAA on the high electron density phantom, 3000 HU.

**Table 7:** Comparison of the features of PDIP and the presented method

*Feature*	*Portal Dosimetry*	*AAA Portal Dose Prediction*
FFF modes	No.	Yes.
Time needed for the verification	Approx. 5 min (plus machine time, depending on number of fields and MU).	Approx. 10 min (plus machine time, depending on number of fields and MU).
Sensibility	Both methods are expected to have similar sensitivity as the acquired image is essentially the same.	
Dose calculation	Most dose calculation errors will go unnoticed.	Leaf modeling and output factors are taken into account.
Leaf movement	Both methods are expected to have similar sensitivity to leaf movement errors.	
Static or wedged fields	No. Machine QA was not built‐in.	Yes. Machine QA capable.
Initial setup	Requires measurements, modeling and verification.	Requires only verification.

## V. CONCLUSIONS

A method for FFF Portal Dosimetry verification was presented. This procedure focuses on FFF modes, but is also applicable to flattened modes too. The method relies on the Portal Dosimetry software, it takes no more than 3 extra min per field (5 for arc), and the rest of the process keeps the standard Portal Dosimetry workflow. The process is simple (once it has been setup) and the verification can be carried out by a radiographer.

The method presented relies on AAA algorithm, so it requires no extra hardware and no additional data for its modeling. The setup of this method is also straightforward. Only a small piece of software is needed (freely available under request, as long as authorship is credited). The fact that the method relies on the clinically used algorithm makes it suitable for the verification of Varian's PDIP algorithm. Also the use of this algorithm makes the verification “more clinical” because the dose calculation is verified (although heterogeneity or uneven contours are not).

Absolute dose verification (the comparison of CU in predicted and measured images) will be the focus of future development.

This method is also useful on machine QA because it is capable of generating a predicted image of a static field (e.g., wedge fields).

The method presented was capable of verifying all the clinical fields treated in our institution with FFF modes and several (randomly chosen) 6X fields.

## ACKNOWLEDGMENTS

The authors wish to acknowledge A. Llewellyn and JC. Belloso (Varian engineers) for their help in the development of this study. Special thanks must be given to N. Seoane (meteorology and data analysis expert from INTA) for her invaluable help and support.

## COPYRIGHT

This work is licensed under a Creative Commons Attribution 3.0 Unported License.
